# Supplementary material for: Bacillus thuringiensis Exopolysaccharide BPS-2 Ameliorates Ulcerative Colitis in a Murine Model Through Modulation of Gut Microbiota and Suppression of the NF-κB Cascade
Source: Foods. 2025 Jul 4;14(13):2378. doi: 10.3390/foods14132378 (PMC12249288; doi:10.3390/foods14132378)
Supplement: Supplementary file 1 [file foods-14-02378-s001.zip › foods-3695946-supplementary.pdf]

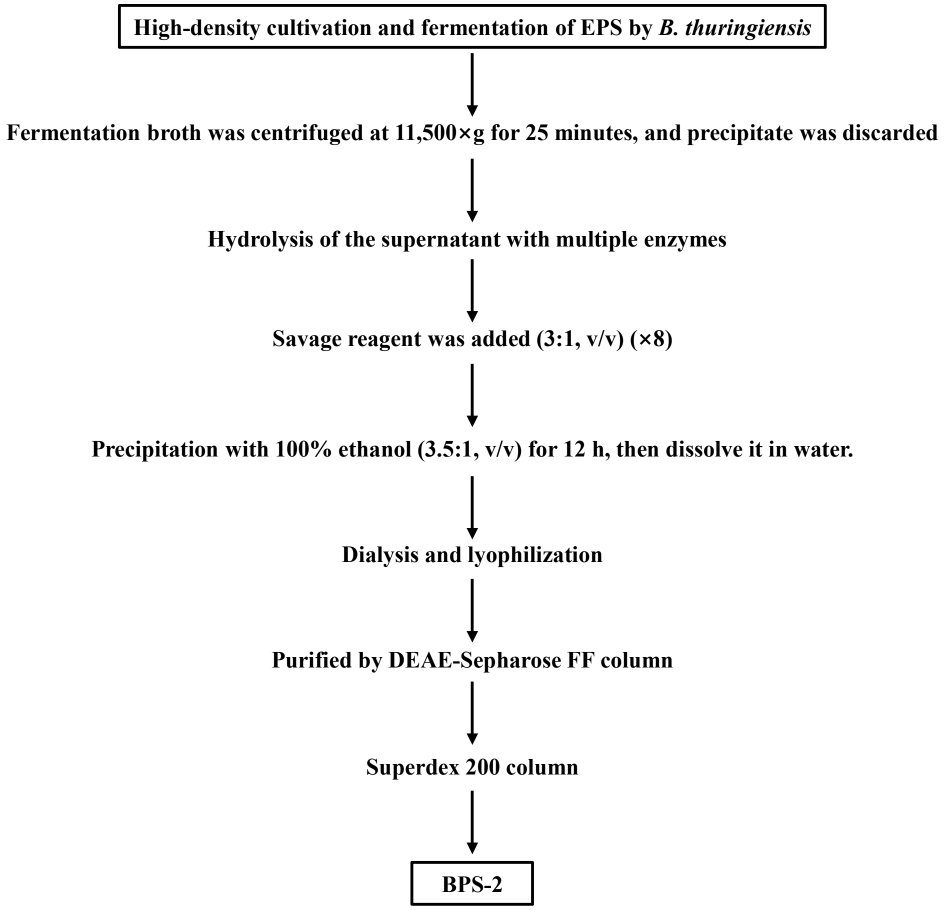

Figure S1. The preparation of BPS-2.

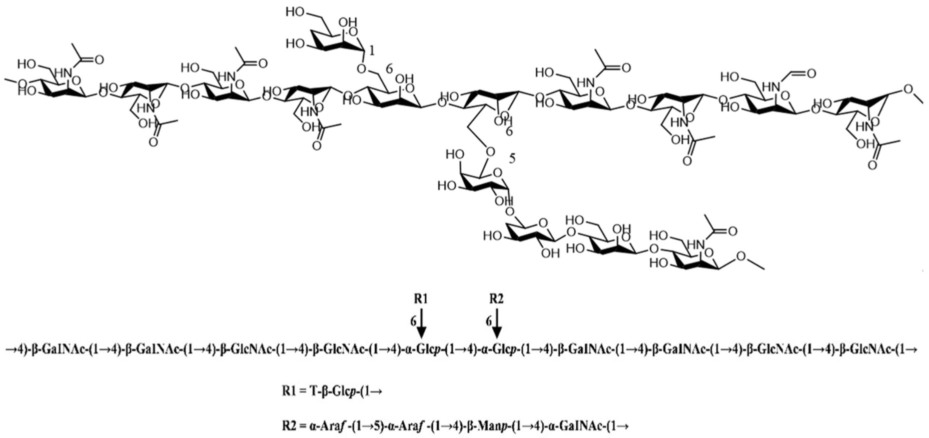

Figure S2. Structure of the base unit of BPS-2. The BPS-2 backbone consists of four 1,4-β-GlcNAc, four 1,4-β-GaINAc, and two 1,4-α-Glc*p* units, with branched chains (one T-β-Glc*p*, one 1,4-β-Man*p*, one 1,6-α-GaINAc, and two 1,5-α-Ara*f*) with 10-11 units forming a polymer.

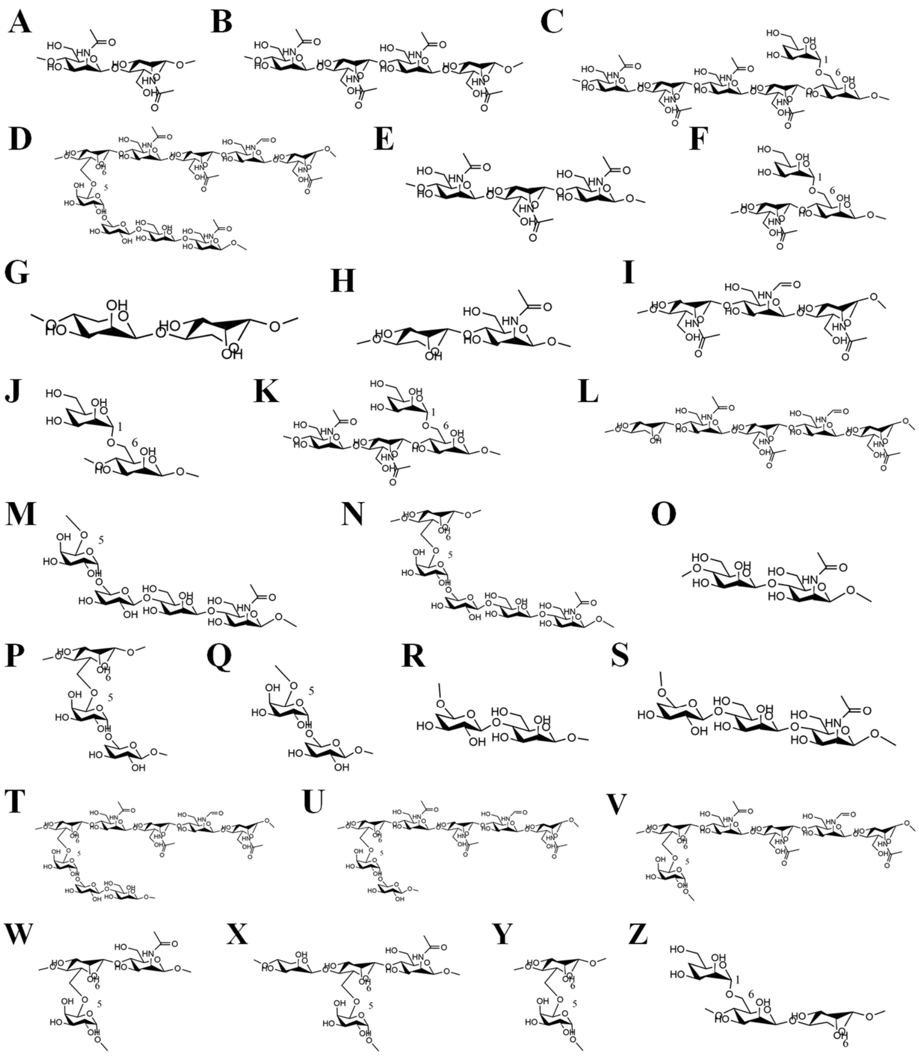

Figure S3. (A–Z) Potential bioactive oligosaccharide fragments in the polysaccharide BPS-2.

Table S1. Disease activity index score.

Weight loss	Fecal occult blood condition	Fecal consistency	Scores
0	No occult blood	Normal	0
1-5%	No occult blood	Somewhat loose	1
6-10%	No occult blood	Loose	2
11-15%	Occult blood present	Highly loose	3
> 15%	Rectal bleeding	Diarrheal stool	4

Table S2. Standard for evaluation of histological injury.

Inflammatory disease	Depth of pathological changes	Crypt destruction	Range of pathological changes	Scores
None	None	None	None	0
Mild	Mucosa layer	1/3	1-25%	1
Moderate	Submucosa layer	2/3	26-50%	2
Severe	Muscularis propria layer	100%	51-75%	3
Extremely severe	Subserosa layer and Serosa layer	Intestinal epithelial destruction	76-100%	4

Table S3. The values of the minimum binding energy, the number of hydrogen bonds, and the information on the specific binding sites between the NF-κB, p50, and p65 subunits and the screened BPS-2 oligosaccharide fragments. The screened BPS-2 oligosaccharide fragments are consistent with those in Figure S3.

Component	Minimum binding energy (Kcal/mol)	Number of hydrogen bonds	Amino acid residues
NF-κB-BPS-2A	-5.6	5	ASN103, ILE104, SER171, VAL169
NF-κB-BPS-2B	-6.6	12	SER45, SER51, LYS195, LYS218, GLY31, ASN186, ARG305
NF-κB-BPS-2C	-6.9	13	LYS49, LYS272, LYS241, SER240, ASN247, LEU248, THR339, ARG246
NF-κB-BPS-2D	-5.4	13	LYS241, ARG54, GLY52, ARG246, ASP271, ASN247, LYS49, LYS77
NF-κB-BPS-2E	-6.0	6	CYS105, TYR100, SER112, ILE110, ARG108
NF-κB-BPS-2F	-6.4	11	SER110, LEU140, LEU111, ASP118, CYS116, GLY113, ARG154
NF-κB-BPS-2G	-5.7	4	ASN103, GLN201, VAL169
NF-κB-BPS-2H	-5.6	4	CYS105, HIS111, SER112, GLN114
NF-κB-BPS-2I	-6.2	4	CYS105, ARG108, ILE110
NF-κB-BPS-2J	-5.6	3	GLN201, SER171
NF-κB-BPS-2K	-6.6	10	LYS93, GLN114, SER112, HIS111, CYS105
NF-κB-BPS-2L	-6.7	12	SER51, GLY31, LYS195, ASP217, ASN186, LYS218, ARG305, GLN306, LYS272
NF-κB-BPS-2M	-6.7	10	GLN128, GLU89, GLY98, LYS122, LYS37
NF-κB-BPS-2N	-7.3	9	LYS102, ASN103, VAL169, HIS105, SER171, GLN201, GLU204
NF-κB-BPS-2O	-5.9	6	SER171, GLN201, ASN103

NF-κB-BPS-2P	-6.3	5	LYS93, GLN114, SER112
NF-κB-BPS-2Q	-6.5	6	GLU204, GLN201, SER171
NF-κB-BPS-2R	-6.0	5	ASN103, SER171, GLN201
NF-κB-BPS-2S	-6.5	7	ASN200, GLU265, ARG201, ARG255
NF-κB-BPS-2T	-6.4	8	SER51, GLY31, ASN186, AGR33, LYS218, LYS195
NF-κB-BPS-2U	-7.6	14	ARG33, AGR30, LYS28, GLU49, ARG50, ARG305, LYS195, SER51
NF-κB-BPS-2V	-6.8	8	ARG305, LYS218, ARG187, ARG33, SER45, GLN114, THR57
NF-κB-BPS-2W	-6.2	7	LEU167, ASN103, LYS102, GLN201, SER171
NF-κB-BPS-2X	-7.0	8	ARG154, ALA108, ASP118, SER110, GLY119
NF-κB-BPS-2Y	-5.9	5	GLY162, GLY180, ILE160, ARG161
NF-κB-BPS-2Z	-6.6	5	ARG184, GLY181, GLY162, ARG161
p50-BPS-2A	-5.6	4	ASN103, ILE104, SER171, VAL169
p50-BPS-2B	-6.6	9	ARG184, ARG161, CYS116, GLY162, GLN177, LYS92, GLY180, PHE225
p50-BPS-2C	-6.6	7	LYS49, ARG51, GLY52, SER240, ASN247
p50-BPS-2D	-6.7	16	THR339, GLU341, ASN247, SER240, GLY52, GLN50, GLN47, LEU45, ILE44
p50-BPS-2E	-6.4	10	GLU179, GLY180, GLN177, GLY162, ARG161, ILE160, ARG184, ASP183
p50-BPS-2F	-6.3	8	ARG154, GLY119, SER110, ASP118
p50-BPS-2G	-5.9	7	ILE104, SER171, ASN103, GLN201
p50-BPS-2H	-5.9	4	GLN201, VAL169, GLY166, SER171
p50-BPS-2I	-6.2	11	ASP118, GLU157, ARG161, ARG184,

			GLY180, GLY181, GLY182, GLY162
p50-BPS-2J	-6.0	5	GLY162, ARG161, PHE225
p50-BPS-2K	-6.2	7	GLU149, ARG154, SER110, ASP118, GLY119, LEU111
p50-BPS-2L	-6.1	8	PHE298, LYS334, PHE295, GLU293, TRP292
p50-BPS-2M	-6.5	11	GLY181, ARG161, GLY162, THR122, LYS92, SER224, GLY223
p50-BPS-2N	-5.7	9	GLY182, GLY180, ARG161, GLY162, LYS92
p50-BPS-2O	-5.9	5	ILE160, GLY181, GLY182, PHE225
p50-BPS-2P	-6.2	7	GLY162, ARG161, PHE225
p50-BPS-2Q	-6.5	6	ILE104, SER171, GLN201
p50-BPS-2R	-6.0	4	ASN103, GLN201, SER171
p50-BPS-2S	-6.5	12	SER171, VAL169, ASN103, GLN201, GLN196
p50-BPS-2T	-5.6	17	GLN50, AGR51, ASP336, GLU338, LEU337, THR339, GLN330, ARG281, TYR283
p50-BPS-2U	-7.1	15	THR122, PHE225, GLY162, ARG161, GLY181, GLY182, ILE160, GLU157
p50-BPS-2V	-7.1	9	ASN244, GLU204, GLN201, ASN103, SER171, VAL169
p50-BPS-2W	-6.4	7	LYS102, GLN201, VAL169, SER171
p50-BPS-2X	-6.6	6	GLN279, PHE295, THR313, PHE298
P50-BPS-2Y	-6.3	4	ARG161, GLY162, GLN177
p50-BPS-2Z	-6.6	5	GLY181, SER224, GLY162, ILE160, ARG184
p65-BPS-2A	-5.5	7	LYS93, SER112, ILE110, ASP103

p65-BPS-2B	-6.4	7	ASN186, SER45, GLN114
p65-BPS-2C	-6.6	10	LEU194, GLU193, ASP53, SER45, SER51, ARG30, LYS28
p65-BPS-2D	-6.7	14	ARG33, SER45, LYS56, GLY31, ASN186, GLU193, LEU194, LYS218, LYS195, ILE196
p65-BPS-2E	-6.0	7	CYS105, ARG108, HIS111, SER112, ILE110
p65-BPS-2F	-6.3	9	LEU116, SER45, GLY44, SER51, THR57
p65-BPS-2G	-6.7	3	ILE224, GLY237
p65-BPS-2H	-5.6	3	CYS105, SER112
p65-BPS-2I	-6.2	7	SER112, HIS111, ILE110, CYS105, ARG108
p65-BPS-2J	-5.7	10	LEU280, ASP277, ARG278, ARG30
p65-BPS-2K	-6.6	11	LYS93, CYS105, HIS111, SER112, THR60, HIS58, GLN114
p65-BPS-2L	-6.8	6	SER45, LYS28, LYS195, LEU280
p65-BPS-2M	-6.6	13	GLU89, GLN119, CYS120, LYS37, LYS122, GLY98, GLN128
p65-BPS-2N	-6.8	11	CYS105, ASP103, ARG108, ILE110, HIS111, SER112, LYS93, TYR100, GLN114
p65-BPS-2O	-5.9	6	LYS93, ASP94, TYR100, GLN114, SER112
p65-BPS-2P	-6.4	8	LYS93, GLN114, CYS105, SER112, HIS111
p65-BPS-2Q	-6.1	7	CYS38, LYS122, GLN119, LYS37, GLN132
p65-BPS-2R	-6.0	5	TYR100, GLN114, SER112, CYS105, ARG108
p65-BPS-2S	-6.4	4	TYR100, SER112, ARG108
p65-BPS-2T	-7.3	16	GLN220, ASN190, ASN155, ASN186, ARG187, GLU193, LYS218, LYS195, SER51
p65-BPS-2U	-7.8	15	ARG50, SER51, LYS28, ARG30, GLU193, ASN186, GLU279, LYS195, ASP217,

			LYS218
p65-BPS-2V	-7.0	15	SER51, ARG50, LYS28, ARG30, ASN186, GLU193, LYS195, ASP217, GLU279, LYS218
p65-BPS-2W	-6.1	4	GLN114, LYS93, SER112
p65-BPS-2X	-6.6	6	SER45, ARG33, GLU193, LYS195
p65-BPS-2Y	-5.8	4	ASP103, CYS105, SER112
p65-BPS-2Z	-6.2	7	ASP103, SER112, CYS105, GLN114, LYS93
